# Asymptomatic apical aneurysm of the left ventricle with intracavitary thrombus: a diagnosis missed by echocardiography

**DOI:** 10.1590/0100-3984.2016.0199

**Published:** 2018

**Authors:** Kamila Seidel Albuquerque, João Maurício Canavezi Indiani, Marcelo Fontalvo Martin, Beatriz Morais e Rodrigues Cunha, Marcelo Souto Nacif

**Affiliations:** 1 Unidade de Radiologia Clínica (URC), São José dos Campos, SP, Brazil; 2 Universidade Federal Fluminense (UFF), Niterói, RJ, Brazil

Dear Editor,

We report the case of a 63-year-old male, with a history of acute myocardial infarction (AMI) and angioplasty 10 years prior, who was asymptomatic at presentation. He stated that he had not undergone routine clinical follow-up and was therefore submitted to echocardiography for functional evaluation. Moderate dilation and dysfunction of the left ventricle (LV) were detected, although with limitation in the evaluation of the apex, without information on the presence of an aneurysm or thrombus. Coronary computed tomography angiography (CCTA) was performed in order to identify in-stent restenosis, and the images showed apparent subocclusion distal to the stent in the anterior descending artery (Figure 1A) and a large aneurysm with parietal thinning in the anterior/anteroseptal medial segments, septal/anterior apical segments, and apex of the LV. It was not possible to detect significant systolic ballooning, because there was a large thrombus lining the intracavitary portion and that was confused with normal wall thickness of the LV. The thrombus had an organized appearance, albeit without signs of calcification, and was markedly hypodense, with a fixed aspect and no contrast enhancement, which had likely made it difficult to identify in the initial (echocardiographic) assessment (Figures 1B and 1C).

**Figure 1:**
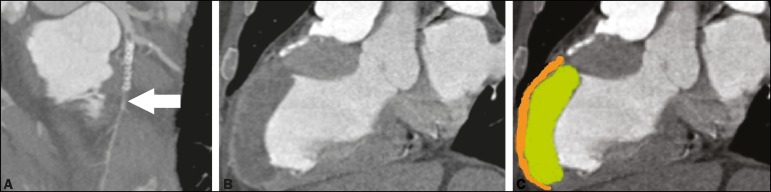
**A:** CCTA with a reconstruction curve showing probable subocclusion downstream of the stent (arrow). **B,C:** Cardiac computed tomography of the heart in the longitudinal axial plane, in a pseudo-two-chamber view, showing the region of the LV aneurysm with marked thinning of the medioapical anterior wall (2 mm thick - orange) and normal thickness in the anterior basal segment. Note the large thrombus simulating normal wall thickness of the LV (green).

Ventricular aneurysm is a serious complication of transmural myocardial infarction (occurring in 5-38% of cases), being the most common mechanical complication, typically evolving to physical limitations and having a negative impact on quality of life^(1-4)^. It is defined as myocardial ventricular wall thinning and dilation, with distinct margins, leading to akinesia or dyskinesia of one or more myocardial segments during ventricular contraction^(1,2-5)^. It typically affects the anteroapical region of the LV, because the blood supply of the anterior wall is highly dependent on the anterior descending artery^(2,3)^. Ventricular aneurysm develops within two to ten days after AMI, becoming apparent in the first year after the infarction, with an incidence of 30-35% in patients who have experienced AMI^(4-6)^. As a secondary finding, intracavitary thrombus affects approximately 40-60% of patients^(4)^ and results from the inflammatory process in the endocardial region affected by the AMI, being associated with the hypokinesia and hypercoagulability existing in the infarction, increasing the risk of a thromboembolic event after the third month in patients with ventricular aneurysm. There is a broad range of symptoms in LV aneurysms, ranging from none to dyspnea, heart failure, or angina, as well as severe manifestations such as acute pulmonary edema, thromboembolism, and ventricular rupture^(5-7)^. In the treatment of severe refractory cases, surgical procedures, such as plication, excision/suture, imbrication, and patch interposition, are indicated^(8)^. In the case presented here, despite the extensive area of left ventricular dyskinesia with aneurysm formation and adherent intracavitary thrombus, the patient remained asymptomatic, an uncommon presentation in large aneurysms, which was diagnosed only through CCTA, a noninvasive method that not only allows the diagnosis to be made but also provides accurate measurements and can be used in the postoperative follow-up^(1,4-6,9-11)^. Routine screening tests, such as echocardiography, often fail to assess the apex of the LV, even with a good access window^(1,2,7)^. In addition to allowing the diagnosis to be made, the CCTA findings promoted patient adherence to the treatment.

## References

[1] Assunção FB, Oliveira DC, Souza VF (2016). Cardiac magnetic resonance imaging and computed tomography in
ischemic cardiomyopathy: an update. Radiol Bras.

[2] Cardoso MB, Azevedo CHNF, Teixeira CO (2001). Aneurisma do ventrículo esquerdo pós-infarto do
miocárdio: correlação da semiotécnica
complementar com os achados anatomopatológicos: relato de quatro
casos com necropsia. Rev Ciênc Méd, Campinas.

[3] Debray M, Pautas E, Dulou L (2001). Aneurysm of the left ventricle: a two-decade silent
history. J Am Geriatr Soc.

[4] Strecker T, Baum U, Harig F (2006). Visualization of a large ventricular aneurysm in a young man by
16-slice multi-detector row spiral computed tomography before successful
surgical treatment. Int J Cardiovasc Imaging.

[5] Achenbach S, Ropers D, Daniel WG (2003). Calcified left ventricular aneurysm. N Engl J Med.

[6] Evangelou D, Letsas KP, Gavrielatos G (2006). Giant left-ventricular pseudoaneurysm following silent myocardial
infarction. Cardiology.

[7] Makaryus AN, Manetta F, Goldner B (2005). Large left ventricular pseudoaneurysm presenting 25 years after
penetrating chest trauma. J Interv Cardiol.

[8] Loures DRR, Carvalho RG, Lima Jr JD (1997). Tratamento cirúrgico dos aneurismas de ventrículo
esquerdo e isquemia coronária. Rev Bras Cir Cardiovasc.

[9] Assunção FB, Oliveira DCL, Souza VF (2016). Cardiac magnetic resonance imaging and computed tomography in
ischemic cardiomyopathy: an update. Radiol Bras.

[10] Rochitte CE (2016). Cardiac MRI and CT: the eyes to visualize coronary arterial
disease and their effect on the prognosis explained by the
Schrödinger's cat paradox. Radiol Bras.

[11] Neves PO, Andrade J, Monção H (2017). Coronary artery calcium score: current status. Radiol Bras.

